# A rare *CACNA1H* variant associated with amyotrophic lateral sclerosis causes complete loss of Ca_v_3.2 T-type channel activity

**DOI:** 10.1186/s13041-020-00577-6

**Published:** 2020-03-06

**Authors:** Robin N. Stringer, Bohumila Jurkovicova-Tarabova, Sun Huang, Omid Haji-Ghassemi, Romane Idoux, Anna Liashenko, Ivana A. Souza, Yuriy Rzhepetskyy, Lubica Lacinova, Filip Van Petegem, Gerald W. Zamponi, Roger Pamphlett, Norbert Weiss

**Affiliations:** 1 Institute of Organic Chemistry and Biochemistry, Czech Academy of Sciences, Flemingovo nam 2, 16610 Prague, Czech Republic; 2 Third Faculty of Medicine, Charles University, Prague, Czech Republic; 3 Center of Biosciences, Institute of Molecular Physiology and Genetics, Academy of Sciences, Bratislava, Slovakia; 4 Department of Physiology and Pharmacology, Cumming School of Medicine, University of Calgary, Calgary, Canada; 5 Department of Biochemistry and Molecular Biology, University of British Columbia, Vancouver, Canada; 6 Discipline of Pathology, Brain and Mind Centre, The University of Sydney, Sydney, NSW Australia

**Keywords:** ALS, Amyotrophic lateral sclerosis, Motor neuron disease, *CACNA1H*, Mutation, Calcium channel, Ca_v_3.2 channel, T-type channel, Biophysics

## Abstract

Amyotrophic lateral sclerosis (ALS) is a neurodegenerative disorder characterized by the progressive loss of cortical, brain stem and spinal motor neurons that leads to muscle weakness and death. A previous study implicated *CACNA1H* encoding for Ca_v_3.2 calcium channels as a susceptibility gene in ALS. In the present study, two heterozygous *CACNA1H* variants were identified by whole genome sequencing in a small cohort of ALS patients. These variants were functionally characterized using patch clamp electrophysiology, biochemistry assays, and molecular modeling. A previously unreported c.454GTAC > G variant produced an inframe deletion of a highly conserved isoleucine residue in Ca_v_3.2 (p.ΔI153) and caused a complete loss-of-function of the channel, with an additional dominant-negative effect on the wild-type channel when expressed in *trans*. In contrast, the c.3629C > T variant caused a missense substitution of a proline with a leucine (p.P1210L) and produced a comparatively mild alteration of Ca_v_3.2 channel activity. The newly identified ΔI153 variant is the first to be reported to cause a complete loss of Ca_v_3.2 channel function. These findings add to the notion that loss-of-function of Ca_v_3.2 channels associated with rare *CACNA1H* variants may be risk factors in the complex etiology of ALS.

## Introduction

Amyotrophic lateral sclerosis (ALS), also known as motor neuron disease or Lou Gehrig’s disease, is a heterogeneous neuromuscular disease characterized by the degeneration of cortical, brain stem and spinal motor neurons that leads to muscle weakness and paralysis. Disease onset averages between 40 and 70 years of age [1], and the annual incidence worldwide is estimated to be between one to three per 100,000 people [2]. ALS is best regarded as a complex genetic disorder with a Mendelian pattern of inheritance in approximately 5–10% of patients (familial ALS, fALS), but most patients have no discernable family history of the disease which is then referred to being “sporadic” or “isolated” in nature (sALS) [3]. However, the observation that established fALS genes are also implicated in sALS makes the distinction between fALS and sALS more abstruse [4]. For instance, mutations in the most common ALS genes (*SOD1*, *FUS*, *TARDBP*, *C9orf72*, *VCP*, and *PFN1*) account for up to 70% of fALS patients and about 10% of sALS [5]. In addition, several genes and loci in apparent sALS patients have been proposed to be associated with an increased risk of ALS, or to modify the onset or progression of the disease, which highlights the importance of genetic risk factors [6]. Among these genes, the most prominent are *ATXN2* [7], *UNC13A* [8], *ANG* [9], and *SMN1* [10]. Recently, whole exome sequence analysis of case-unaffected-parents trios identified two compound heterozygous recessive missense mutations in the gene *CACNA1H* [11, 12].

In the present study, we report two additional *CACNA1H* variants (c.3629C > T, p.P1210L and c.454GTAC > G, p.ΔI153) identified using whole genome sequencing of a cohort of 34 sALS patients, with sequencing undertaken at the Genome Institute, Washington University, St Louise USA. The method of whole genome analysis was the same as that reported in a separate study [11]. Whole genome analyses reveal no pathogenetic single nucleotide or structural differences between monozygotic twins discordant for amyotrophic lateral sclerosis [13]. No unaffected parent DNA was subjected to whole genome sequencing, so it was not possible to determine if the variants were recessive or de novo in nature [11]. Functional analysis of these two variants revealed a complete loss of Ca_v_3.2 channel function associated with the ΔI153 variant and a dominant-negative effect of this variant on the wild-type channel when expressed in *trans*.

## Results

### Whole genome sequencing identifies heterozygous CACNA1H mutations in ALS patients

**Fig. 1:**
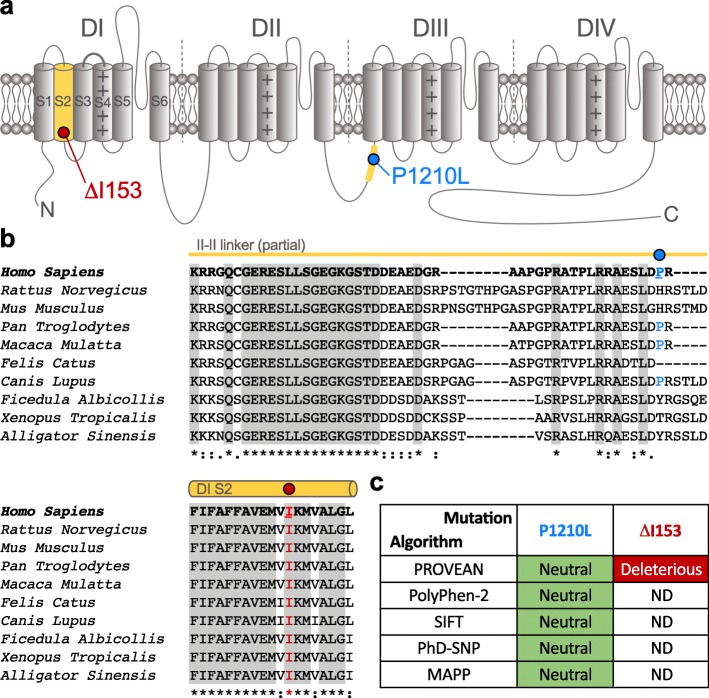
Location of ALS-associated Ca_v_3.2 variants. **a** Schematic representation of the membrane topology of Ca_v_3.2 depicting the position of the ΔI153 (red circle) and P1210L variants (blue circle). **b** Amino acid sequence alignment of Ca_v_3.2 regions containing the two mutations across several species showing the conservation of the I153 residue. Alignments were performed using UniProt (*Homo sapiens* O95180; *Rattus norvegicus* Q9EQ60; *Mus musculus* O88427; *Pan troglodytes* H2QA94; *Macaca mulatta* A0A1D5R8A8; *Felis catus* M3WP54; *Canis lupus* F1PQE5; *Ficedula albicollis* U3KGY9; *Xenopus Tropicalis* F6U0H3; *Alligator sinensis* A0A3Q0GL31). **c** In silico prediction of the potential impact of the P1210L and ΔI153 mutations on the functioning of Ca_v_3.2 channel

In a previous study, using case-unaffected parents trio exome analyses, we reported an ALS patient with two heterozygous *CACNA1H* missense mutations causing a partial loss-of-function of Ca_v_3.2 channel, suggesting that rare *CACNA1H* variants may represent a risk factor for ALS [11, 12]. In the present study, using whole genome sequencing of a small cohort of ALS patients, we identified two additional heterozygous variants in *CACNA1H*. The first variant (c.3629C > T, p.P1210L) was identified in a man with ALS onset aged 55 years who died aged 62 years. He had no family history of ALS, though his father had Alzheimer’s disease and his mother bipolar disorder. The P1210L variant is located in a non-conserved region of the intracellular linker connecting transmembrane domains II and III (II-III loop) of Ca_v_3.2 (Fig. 1a and b). This variant has previously been reported in 188 out of 240,876 individuals in the gnomAD database (https://gnomad.broadinstitute.org/), including 144 of 193,008 alleles only from individuals who were not ascertained for having a neurological condition in a neurological case/control study. Furthermore, in silico analysis predicted the amino acid change to be neutral (Fig. 1c), suggesting that this variant is likely to not have a major pathological role. The second variant (c.454GTAC > G, p.ΔI153) was identified in a man with ALS onset aged 53 years who died aged 54 years. Although he had no family history of ALS, his mother developed insulin-dependent diabetes mellitus and narcolepsy, and his father presented with early onset dementia, a condition known to precede motor impairment in some people with ALS [14]. This mutation produces an inframe deletion of the isoleucine 153 located in the second transmembrane helix of Ca_v_3.2, a region highly conserved across Ca_v_3.2 channel orthologs (Fig. 1a and b). The ΔI153 variant has only been reported in 1 out of 198,036 individuals in the gnomAD database and this deletion was predicted to be deleterious on the channel (Fig. 1c). Hexanucleotide repeat number in C9orf72, the most common genetic cause of ALS, was normal in both patients.

### The ΔI153 mutation causes a complete loss of Ca_v_3.2 function

**Fig. 2:**
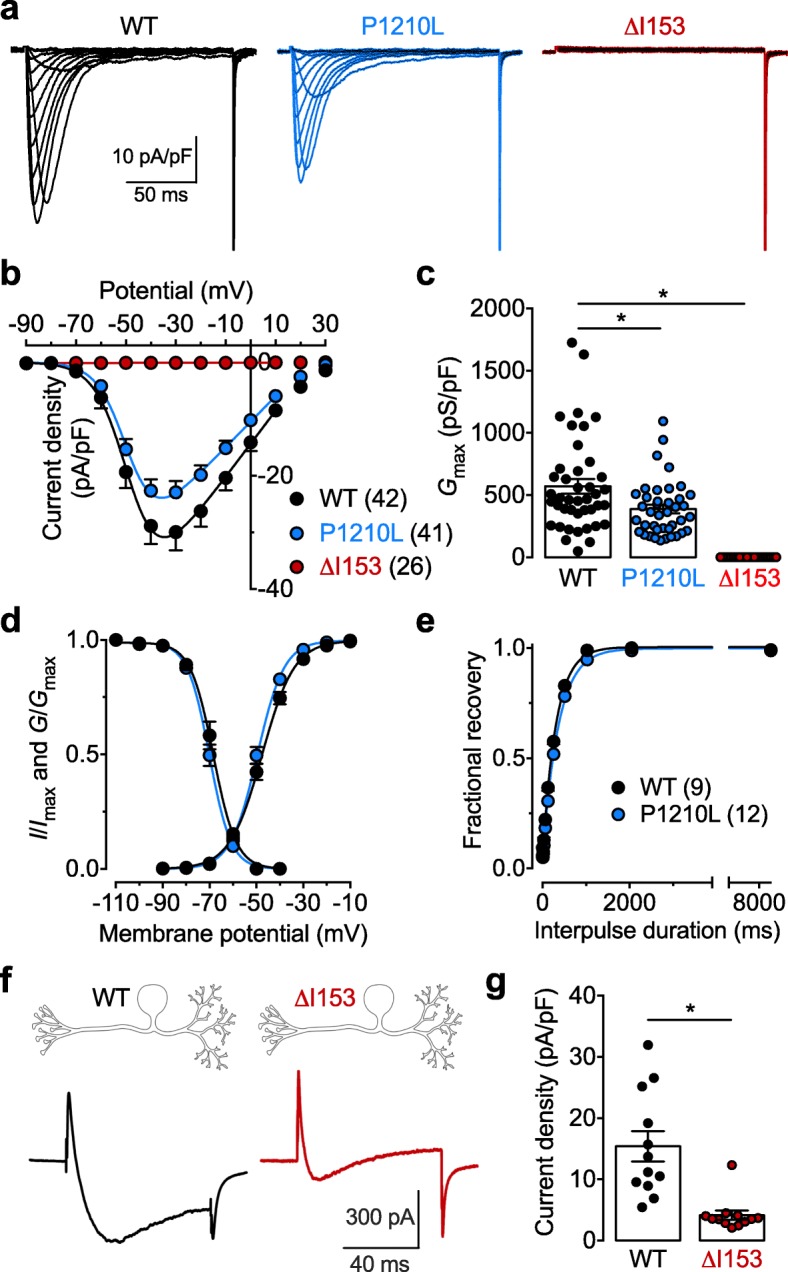
Electrophysiological characterization of Ca_v_3.2 P1210L and ΔI153 variants. **a** Representative T-type current traces recorded in response to 150 ms depolarizing steps to values ranging between − 90 mV and + 30 mV from a holding potential of − 100 mV for wild-type (WT, black traces), P1210L (blue traces), and ΔI153 (red traces) channel variants expressed in tsA-201 cells. **b** Corresponding mean current-voltage relationship (*I*/*V*) for WT (black circles), P1210L (blue circles), and ΔI153 (red circles) channels. **c** Corresponding mean maximal macroscopic conductance (*G*_max_) obtained from the fit of the I/V curves with the modified Boltzmann eq. (1). **d** Mean normalized voltage-dependence of activation and inactivation for WT (black circles) and P1210L channels (blue circles). **e** Mean normalized recovery from inactivation kinetics. **f** Representative T-type current traces recorded from WT (black trace) and ΔI153 DRG neurons (red trace) 3 days after editing of *Cacna1h* by CRISPR/Cas9 in response to 80 ms depolarizing steps to − 25 mV from a holding potential of − 90 mV. **g** Corresponding mean peak T-type current density at − 25 mV in WT and ΔI153 mutant DRG neurons

In the first series of experiments we assessed the functional expression of Ca_v_3.2 P1210L and ΔI153 channel variants expressed in tsA-201 cells by whole-cell patch clamp electrophysiology. Cells expressing the P1210L channel variant displayed a characteristic low-threshold voltage-activated T-type current (Fig. 2a and b) that only differed from cells expressing the wild-type (WT) channel by a 32% reduction (*p* = 0.0125, Mann-Whitney test) of the maximal conductance (*G*_max_) (from 571.3 ± 58.4 pS/pF, *n* = 42 to 387.7 ± 33.9 pS/pF, *n* = 41) (Fig. 2c). The main electrophysiological properties, including voltage-dependence of activation and inactivation (Fig. 2d), and recovery from inactivation (Fig. 2e), remained unaffected. In cells expressing the ΔI153 channel variant, we did not record any T-type conductance (Fig. 2a-c). It is noteworthy that experimental conditions known to favor the expression of misfolded proteins, such as treatment of cells with the proteasome inhibitor MG132 or decrease of cell incubation temperature to 30 °C, were used but failed to restore a T-type conductance. Additionally, co-expression of the ΔI153 channel variant with Stac1 or with a calnexin-derived peptide that has previously been reported to potentiate the expression of Ca_v_3.2 in the plasma membrane [15, 16] also failed to restore T-type currents (data not shown). The lack of functional expression of the ΔI153 channel variant could have been inherent in our experimental conditions using recombinant channels, so we aimed to further assess the phenotypic effect of the ΔI153 mutation on native Ca_v_3.2 channels in a neuronal environment. Therefore, we used a CRISPR/Cas9 approach to introduce the ΔI153 mutation in native Ca_v_3.2 channels in cultured dorsal root ganglion (DRG) neurons. DRG neurons were used as a model system since these neurons are known to display a T-type conductance that is almost exclusively carried by Ca_v_3.2 channel subtype [17]. Consistent with our observation with recombinant Ca_v_3.2 channels, T-type currents recorded from Ca_v_3.2 ΔI153 DRG neurons 3 days after gene editing were reduced by 73% (Mann-Whitney *p* < 0.0001) compared to wild type neurons (from 15.4 ± 2.5 pA/pF, *n* = 12 to 4.1 ± 0.8 pA/pF, *n* = 12) (Fig. 2f and g).

Collectively, these data revealed a mild loss of channel function associated with the P1210L variant, and the deleterious effect of the ΔI153 mutation leading to a complete loss of Ca_v_3.2 activity.

### The ΔI153 mutation disrupts Ca_v_3.2 biogenesis

**Fig. 3:**
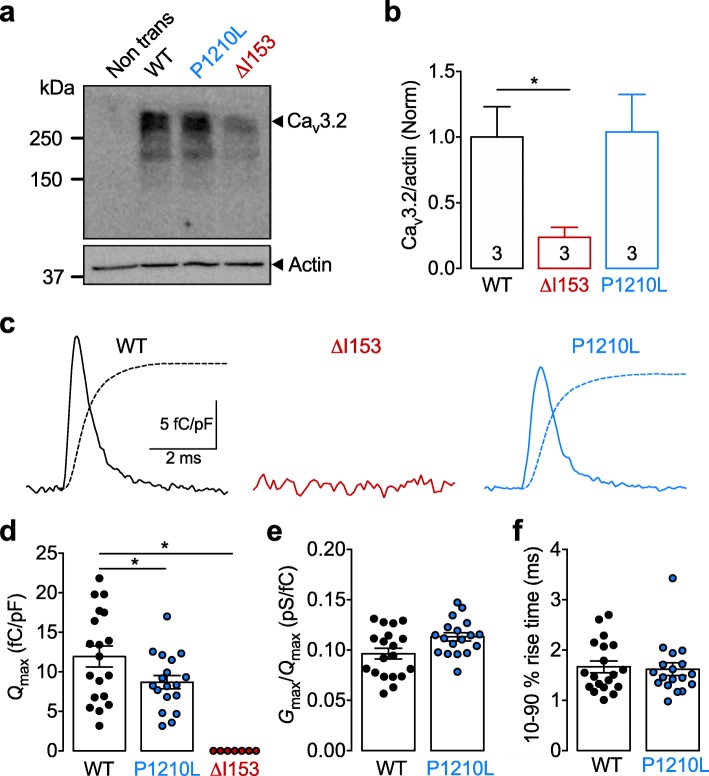
Expression of Ca_v_3.2 P1210L and ΔI153 variants. **a** Representative immunoblot of Ca_v_3.2 from tsA-201 cells expressing wild-type (WT), P1210L, and ΔI153 channel variants. **b** Corresponding mean expression levels of P1210L and ΔI153 variants relative to WT channels. **c** Representative charge movement traces recorded at the ionic reversal potential from cells expressing wild-type (WT, black trace), P1210L (blue trace), and ΔI153 (red trace) channel variants. The dotted line depicts the time course of the integral for each trace. **d** Corresponding mean *Q*_max_ values calculated for each investigated cell. **e** Corresponding mean *G*_max_/*Q*_max_ ratios. **f** Corresponding mean 10–90% rise times calculated from the integral time course shown in panel **c**

The alteration of T-type currents in ALS-associated Ca_v_3.2 variants could originate from an overall decreased expression of channel proteins, reduced channel density in the plasma membrane, altered gating of the channel, or from a combination of several of these. Therefore, we first assessed the expression levels of P1210L and ΔI153 channel variants in tsA-201 cells by western blot (Fig. 3a). Immunoblot analysis from total cell lysates showed that the P1210L channel variant was present at a similar level as the WT channel (Fig. 3b). In contrast, the expression level of the ΔI153 channel variant was reduced by 78% (Mann-Whitney *p* = 0.0286), suggesting that this variant may undergo extensive degradation (Fig. 3b). Next, we aimed to assess the expression of Cav3.2 channel variants at the cell surface. Therefore, we analyzed charge movements (*Q*) that refer to the movement of the channel voltage-sensor in the plasma membrane in response to electrical membrane depolarizations. Total charges (*Q*_max_) were assessed at the reversal potential of the ionic current, where we can consider *Q*_rev_ to be equal to *Q*_max_, providing an accurate assessment of the total number of channels in the plasma membrane (Fig. 3c). In cells expressing the P1210L variant, we observed a 27% reduction of *Q*_max_ (t-test *p* = 0.0467) compared to cells expressing the WT channel (from 12.0 ± 1.3 fC/pF, *n* = 19 to 8.7 ± 0.8 fC/pF, *n* = 18) (Fig. 3d). This reduction of *Q*_max_ is similar to the reduction of the maximal T-type conductance we previously observed (32%, Fig. 2c), suggesting that the decrease of the T-type conductance in cells expressing the P1210L channel variant is likely caused by a reduced expression of the channel in the plasma membrane. This notion is further supported by the observation that neither the *G*_max_/*Q*_*max*_ dependency (Fig. 3e), nor the kinetics of charge movements (Fig. 3f), were modified, indicating that the gating properties of the P1210L channel variant remained unaltered. In contrast, we did not detect any charge movement in cells expressing the ΔI153 channel variant (Fig. 3c and d), suggesting that despite being biochemically expressed, this variant is not present in the plasma membrane.

Altogether, these data are consistent with a mildly decreased surface expression of the P1210L variant without additional alterations. Importantly, these data demonstrate the profound deleterious effect of the ΔI153 mutation on the biogenesis and surface trafficking of Ca_v_3.2 channels.

### Dominant-negative effect of the ΔI153 channel variant

**Fig. 4:**
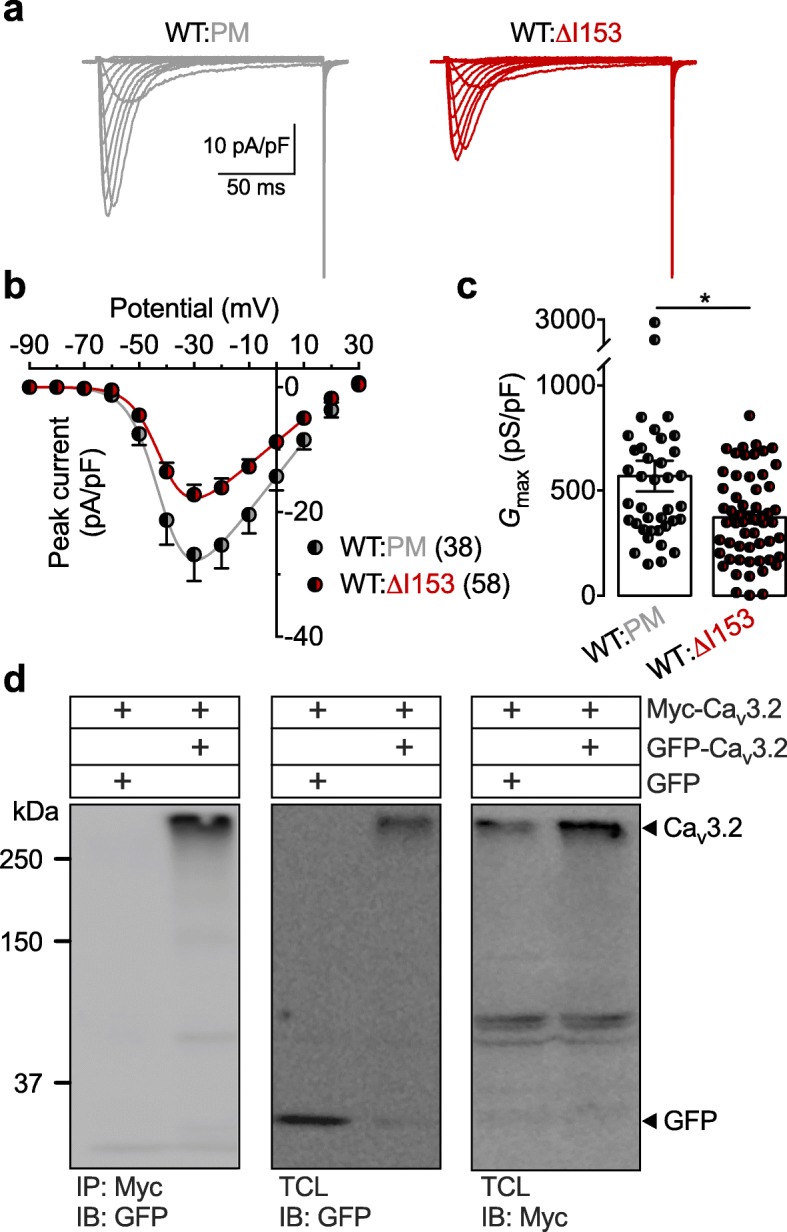
Electrophysiological characterization of Cav3.2 WT and ΔI153 expressed in *trans*. **a** Representative T-type current traces recorded from tsA-201 cells expressing WT channels in combination with either the ΔI153 variant (WT:ΔI153, red traces) or the cation-impermeant but trafficking-competent Ca_v_3.2 pore mutant (WT:PM; grey traces) in a ratio 1:1. **b** Corresponding mean current-voltage relationship (*I*/*V*) for WT:ΔI153 (black|red circles), and WT:PM (black|grey circles) conditions. **c** Corresponding mean maximal macroscopic conductance (*G*_max_). **d** Co-immunoprecipitation of Ca_v_3.2 from tsA-201 cells co-transfected with a Myc-tagged and GFP-tagged Ca_v_3.2. The left panel shows the result of the co-immunoprecipitation of Myc-Ca_v_3.2 with GFP-Ca_v_3.2 using an anti-Myc antibody. The middle and right panels show the immunoblot of GFP-Ca_v_3.2 and Myc-Ca_v_3.2 using and anti-GFP and anti-Myc antibody, respectively

Given the heterozygosity of the ΔI153 mutation and the defective trafficking of the ΔI153 channel variant, we aimed to test whether this variant could have a dominant-negative effect on WT channels. Therefore, we co-expressed the WT and ΔI153 channels in tsA-201 cells in a 1:1 ratio (equal amount of cDNAs) and compared T-type currents with cells expressing the WT channel in combination with a cation-impermeant but trafficking-competent channel (PM). Recording of T-type currents in cells expressing a combination of WT:ΔI153 channels (Fig. 4a) revealed a 35% reduction (Mann-Whitney *p* = 0.0080) of the maximal T-type conductance compared to cells expressing a combination of WT:PM channels (from 569 ± 73 pS/pF, *n* = 38 to 372 ± 27 pS/pF, *n* = 58) (Fig. 4b and c), indicating that the ΔI153 variant produced a dominant-negative effect on the WT channel when expressed in *trans*. In contrast, the voltage-dependence of activation and inactivation remained unaltered. Given the comparatively mild phenotype produced by the P1210L mutation, the P1210L variant was not tested in combination with the WT channel. Finally, to test whether this dominant-negative effect could be mediated by an interaction between Ca_v_3.2 subunits, we performed co-immunoprecipitations from tsA-201 cells co-expressing Myc-tagged and GFP-tagged Ca_v_3.2 to discriminate between the two channels. We observed that the GFP-tagged Ca_v_3.2 was immunoprecipitated with the Myc-tagged Ca_v_3.2 using a specific anti-Myc antibody, revealing the ability of Ca_v_3.2 channels to dimerize (Fig. 4d).

Collectively, these data revealed the dominant-negative effect of the ΔI153 variant on the WT channel, a phenomenon likely to be mediated by the interaction between Ca_v_3.2 subunits.

## Discussion

While several common genes are implicated in familial ALS, the occurrence of rare genetic variants in patients with no family history of the disease has emerged as a potential contributing factor in sporadic ALS [11]. In this study, we report two heterozygous *CACNA1H* variants identified by whole genome sequencing of a small cohort of ALS patients. Functional analysis revealed mild to severe alterations of Ca_v_3.2 variants that were consistent with a loss-of-function of the channels.

The P1210L missense mutation was located in a variable region of Ca_v_3.2 and was not predicted to be deleterious. Our electrophysiological analysis showed a moderate reduction of the expression of the P1210L channel variant at the cell surface and an associated reduction in the T-type conductance. We cannot entirely rule out the possibility that the phenotypic expression of the P1210L variant could have differed when introduced into a different Ca_v_3.2 splice variant [18], or when functionally assessed under different experimental conditions [19], but our experimental data together with the relatively high occurrence of this variant in the general population strongly suggest that it is indeed unlikely to be pathogenic. In contrast, the ΔI153 variant had never been reported and was predicted to be deleterious. Electrophysiological analysis revealed a complete loss of functional expression of the ΔI153 variant, and recording of charge movements suggested that this variant was absent from the cell surface. Furthermore, our biochemical analysis revealed a dramatic decrease of the expression level of the channel protein, suggesting that this variant may have undergone extensive degradation. Of particular importance was the dominant-negative effect produced by the ΔI153 variant on the WT channel when the two channels were expressed in *trans*. This effect was likely to be mediated by the ability of Ca_v_3.2 subunits to dimerize, which could have prevented the proper trafficking of the WT channel to the cell surface in the presence of the impaired ΔI153 variant. In this regard, it is worth considering that this dominant-negative effect may also have an effect on other ion channels. Indeed, Ca_v_3.2 channels are known to biochemically interact with several calcium- and voltage-activated potassium and sodium channel subunits [20–23] whose surface trafficking and activity could be affected by the Ca_v_3.2 ΔI153 variant.

**Fig. 5:**
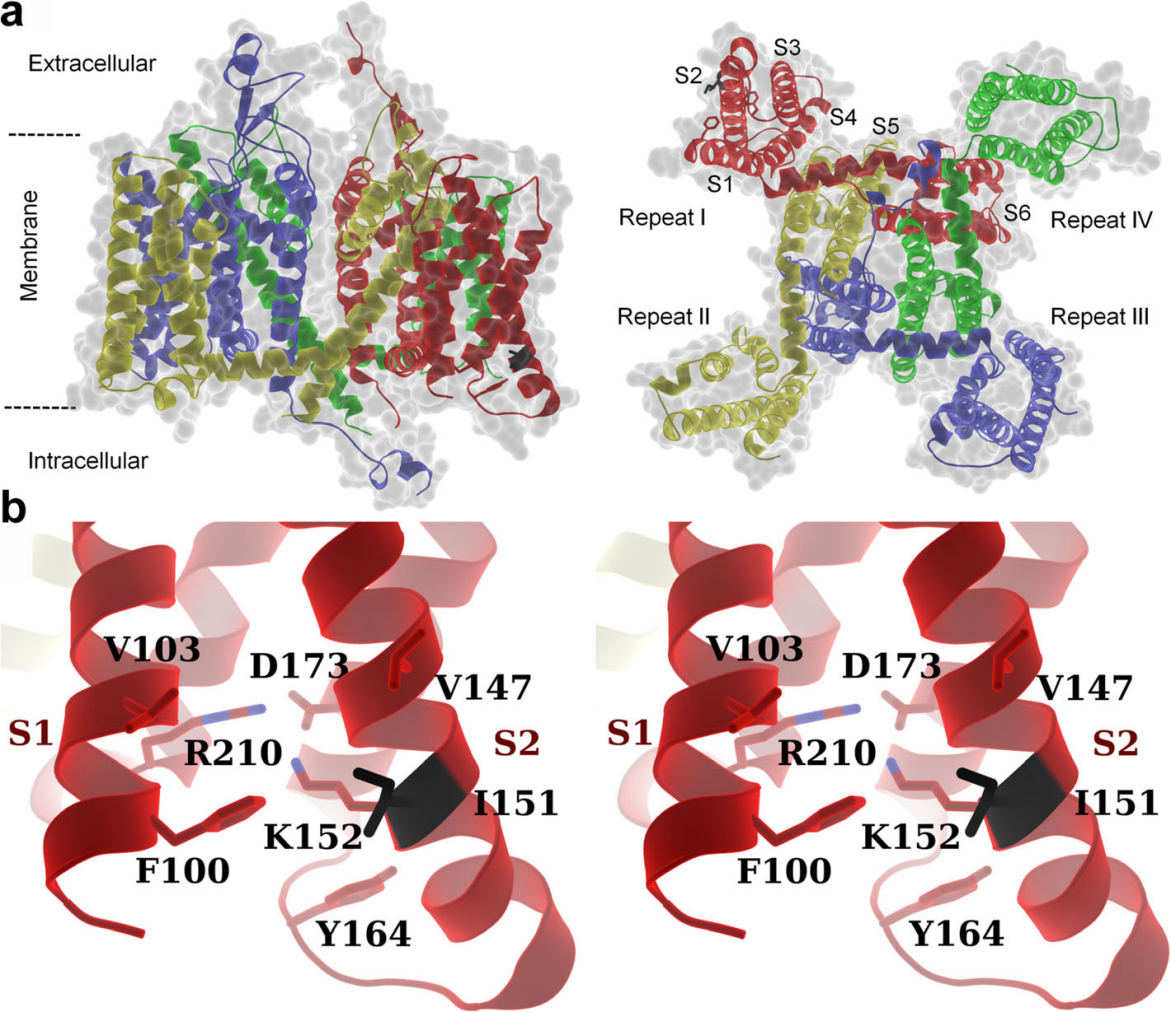
Homology model of human Ca_v_3.2. **a** Cartoon representation of secondary structural elements of human Ca_v_3.2 (Uniprot O95180) homology model (residues 97–1974) based on Ca_v_3.1 (PDB: 6KZO), showing side (left panel) and bottom (right panel) views of the channel. The four domains of Ca_v_3.2 are colored in red, yellow, blue and green. The S1-S6 helices are indicated in red for domain I. Some of the flexible loops connecting the transmembrane helices are not shown, or could not be modeled, due to poor model accuracy or lack of structural information, respectively. The isoleucine 153 (Ile153) is shown in black. **b** Stereo diagram of Ile153 and nearby hydrophobic residues showing its involvement in the helical packing

The molecular mechanisms underlying the deleterious effect of the ΔI153 variant can be appreciated by examining the 3-dimensional environment of I153, and the possible impact of its deletion in the homology model of Ca_v_3.2 we have developed, using the 3.3 Å CryoEM structure of Ca_v_3.1 [24]. In this model, I153 is located within the transmembrane S2 alpha helix of domain I (Fig. 5a), where it is surrounded by hydrophobic residues near the membrane-cytosol interface (Fig. 5b). The nearby hydrophobic residues are highly conserved between L- and T-type channels and I153 shows a clear involvement in the helical packing (Fig. 5b). Therefore, deletion of I153 that results in a net loss of hydrophobicity within the transmembrane segment is likely to alter helix packing in domain I which would result in the misfolding of the channel. Additionally, deletion of I153 would also affect downstream residues in the helix due to a change in the helical register, thus further affecting the helical packing in the voltage-sensing domain.

From a clinical point of view, the loss-of-channel function associated with the ΔI153 variant could have several pathological implications. First, Ca_v_3.2 is present in several central neurons, including reticular thalamic neurons [25], where they contribute to NMDA receptor-mediated synaptic transmission [26]. Given that gain-of-function mutations associated with childhood absence epilepsy were shown to enhance synaptic activities [26], the reciprocal theory would suggest that loss-of-channel function could, in contrast, decrease synaptic transmission. Along these lines, neuroimaging studies have revealed decreased thalamic activity in ALS [27–32], and a recent MRI study reported alterations of thalamic connectivities that mirrored the progressive motor functional decline in ALS [33]. Second, although the functional expression of Ca_v_3.2 in mammalian motor neurons remains elusive, several studies suggest that T-type channels may have a functional role. For instance, Ca_v_3.1 channels are present in turtle spinal motor neurons where they contribute to cellular excitability [34]. In addition, a low-threshold voltage-activated calcium conductance was reported at nodes of Ranvier in mouse spinal motor neurons, suggesting the presence of T-type channels [35]. Third, a T-type channel ortholog is present in motor neurons of the nematode *C. elegans* [36] where it contributes to motor-related functions [37, 38]. Finally, a recent study documented the role of T-type channels in the maintenance of neuronal progenitor cells [39]. A loss-of-function of Ca_v_3.2 could compromise the architecture of nerve cells and precipitate neuronal degeneration.

In conclusion, this newly identified ΔI153 variant is the first to be reported to cause a complete loss of Ca_v_3.2 channel function [40]. Although its pathogenic role in the context of ALS remains to be established, these findings add to the notion that rare *CACNA1H* variants represent a risk factor for ALS. Furthermore, several T-type channels blockers are currently being used for the treatment of epilepsy [41]. The question then arises as to whether long term use of these molecules may present a risk to the development of ALS. This notion should be given particular attention, especially considering that several other T-type channel blockers are currently evaluated in clinical trials for the management of epilepsy and chronic pain symptoms.

## Methods

### Plasmids cDNA constructs and site-directed mutagenesis

The Ca_v_3.2 P1210L and ΔI153 channel variants were created by introducing the respective mutations into the human wild-type HA-tagged Ca_v_3.2 in pcDNA3.1 [42] by PCR-based site-directed mutagenesis using Q5® Site-Directed Mutagenesis Kit (New England Biolabs) and the following mutagenic primers: delI153: 5′-TCAAGATGGTGGCCTTGG-3′ (forward) and 5′-CCATCTCCACCGCAAAAAAG-3′ (reverse); P1210L: 5′-GCCGCCCTCCtGCCTACCAAGTGC-3′ (forward) and 5′-CGGCCGCAGGGGCCGTGG-3′ (reverse). The cation-impermeant Ca_v_3.2 channel was generated by replacing the glutamic acid 378 in domain I with a lysine (E378K) by site-directed mutagenesis. Final constructs were verified by sequencing of the coding sequence of the plasmid cDNAs.

### Cell culture and heterologous expression

Human embryonic kidney tsA-201 cells were grown in DMEM medium supplemented with 10% fetal bovine serum and 1% penicillin/streptomycin (all media purchased from Invitrogen) and maintained under standard conditions at 37 °C in a humidified atmosphere containing 5% CO_2_. Heterologous expression of Ca_v_3.2 channels was performed by transfecting cells with 5 μg plasmid cDNAs encoding for Ca_v_3.2 channel variants using the calcium/phosphate method. For experiments aiming at investigating the dominant negative effect of the ΔI153 variant, cells were co-transfected with 2.5 μg plasmid cDNA encoding for WT channels with either 2.5 μg plasmid cDNA encoding for the ΔI153 channel variant or 2.5 μg plasmid cDNA encoding for a non-conducting but trafficking-competent Ca_v_3.2 (PM).

### Patch clamp electrophysiology

Patch clamp recordings of T-type currents in tsA-201 cells expressing Ca_v_3.2 channel variants were performed 72 h after transfection in the whole-cell configuration at room temperature (22-24 °C) as previously described [43]. The bath solution contained (in millimolar): 5 BaCl_2_, 5 KCl, 1 MgCl_2_, 128 NaCl, 10 TEA-Cl, 10 D-glucose, 10 4-(2-hydroxyethyl)-1-piperazineethanesulfonic acid (HEPES) (pH 7.2 with NaOH). Patch pipettes were filled with a solution containing (in millimolar): 110 CsCl, 3 Mg-ATP, 0.5 Na-GTP, 2.5 MgCl_2_, 5 D-glucose, 10 EGTA, and 10 HEPES (pH 7.4 with CsOH), and had a resistance of 2–4 MΩ. Recordings were performed using an Axopatch 200B amplifier (Axon Instruments) and acquisition and analysis were performed using pClamp 10 and Clampfit 10 software, respectively (Axon Instruments). The linear leak component of the current was corrected online and current traces were digitized at 10 kHz and filtered at 2 kHz. The voltage dependence of activation of Ca_v_3.2 channels was determined by measuring the peak T-type current amplitude in response to 150 ms depolarizing steps to various potentials applied every 10 s from a holding membrane potential of − 100 mV. The current-voltage relationship (I/V) curve was fitted with the following modified Boltzmann eq. (1):

1 $$ I(V)= Gmax\ \frac{\left(V-V\mathrm{rev}\right)}{1+\exp \frac{\left(V0.5-V\right)\ }{k}} $$

with *I*(*V*) being the peak current amplitude at the command potential *V*, *G*_max_ the maximum conductance, *V*_rev_ the reversal potential, *V*_0.5_ the half-activation potential, and *k* the slope factor. The voltage dependence of the whole-cell Ba^2+^ conductance was calculated using the following modified Boltzmann eq. (2):

2 $$ G(V)=\frac{Gmax}{1+\exp \frac{\left(V0.5-V\right)\ }{k}} $$

with *G*(*V*) being the Ba^2+^ conductance at the command potential *V*.

The voltage dependence of the steady-state inactivation of Ca_v_3.2 channels was determined by measuring the peak T-type current amplitude in response to a 150 ms depolarizing step to − 20 mV applied after a 5 s-long conditioning prepulse ranging from − 120 mV to − 30 mV. The current amplitude obtained during each test pulse was normalized to the maximal current amplitude and plotted as a function of the prepulse potential. The voltage dependence of the steady-state inactivation was fitted with the following two-state Boltzmann function (3):

3 $$ I(V)=\frac{Imax}{1+\exp \frac{\left(V-V0.5\right)\ }{k}} $$

with *I*_max_ corresponding to the maximal peak current amplitude and *V*_0.5_ to the half-inactivation voltage.

The recovery from inactivation was assessed using a double-pulse protocol from a holding potential of − 100 mV. The cell membrane was depolarized for 2 s at 0 mV (inactivating prepulse) to ensure complete inactivation of the channel, and then to − 20 mV for 150 ms (test pulse) after an increasing time period (interpulse) ranging between 0.1 ms and 2 s at − 100 mV. The peak current from the test pulse was plotted as a ratio of the maximum prepulse current versus interpulse interval. The data were fitted with the following single-exponential function (4):

4 $$ \frac{I}{Imax}=A\times \left(1-\mathit{\exp}\frac{-t}{\tau}\right) $$

where τ is the time constant for channel recovery from inactivation.

### Measurement of charge movements

Recording of charge movements was performed 72 h after transfection as previously described [44, 45]. The bath solution contained (in millimolar): CsCl 95; TEACl 40, BaCl_2_ 5; MgCl_2_ 1; HEPES 10; glucose 10; pH 7.4 (adjusted with CsOH). Patch pipettes had a resistance ranging from 1.8 MΩ to 2.2 MΩ when filled with a solution containing (in millimolar): CH_3_SO_3_Cs 130; Na-ATP 5; TEACl 10; HEPES 10; EGTA 10; MgCl_2_ 5; pH 7.4 (adjusted with CsOH). Osmolarity of the intracellular solution was approximately 300 mOsmol/L. Osmolarity of the extracellular solution was adjusted by adding sucrose so that the final value was about 2–3 mOsmol/L lower than the osmolarity of the corresponding intracellular solution. Recordings were performed using HEKA EPC10 amplifier (HEKA Electronics). Acquisition and analysis were performed using Patchmaster v90.2 and Fitmaster v2x73.1 and Origin Pro 2015 software, respectively. Only cells with an input resistance less than 5 MΩ were considered. The input resistance and capacity transients were compensated by up to 70% with in-built circuits of the EPC 10 amplifier. Remaining artifacts were subtracted using a -P/8 procedure. ON-gating currents were recorded in response to a series of 5 depolarizing pulses at the reversal potential of the ionic current assessed for each cell, and total gating charge Q_ON_ was calculated as the integral of area below the averaged current traces.

### CRISPR/Cas9 genome editing in DRG neurons

Male rats (6-week-old) were purchased from Charles River and DRG neurons were harvested as described previously [46]. The next day, neurons were transfected with Crispr-Cas9 plasmids (Cas9-sgRNA plasmid and donor plasmid purchased from GeneCopoeia) using Lipofectamine 2000 from Invitrogen (Cat. 11,668–019). The sequence of Crispr RNA was CGTGGAGATGGTGATCAAGA. The donor plasmid contained the homologous arms of the genomic DNA without I153. Whole-cell voltage-clamp recordings of T-type currents were performed 3 days post transfection. The external solution contained (in mM): 40 TEACl, 65 CsCl, 20 BaCl_2_, 1 MgCl_2_, 10 HEPES, 10 D-glucose, pH 7.4. The internal solution contained (in mM): 140 CsCl, 2.5 CaCl_2_, 1 MgCl_2_, 5 EGTA, 10 HEPES, 2 Na-ATP, 0.3 Na-GTP, pH 7.3. We used GFP fluorescence to specifically identify neurons that were transfected with the CRISPR plasmids. The overall percentage of GFP positive neurons in a dish was relatively low, and hence we cannot use bulk genomic sequencing for verification. However, given the large functional effect on current densities, we are confident that the use of GFP fluorescence is an appropriate means of identifying neurons that were targeted with these plasmids. We specifically targeted medium diameter neurons for our analysis. The mean capacitance of the neurons that we recorded from was 24.79 ± 4.40 pF for control neurons versus 21.89 ± 1.31 pF for CRISPR-edited neurons.

### SDS-PAGE and immunoblot analysis

Immunoblot of HA-tagged Ca_v_3.2 channel was performed as previously described [16]. Briefly, total cell lysate from tsA-201 cells expressing HA-Ca_v_3.2 channels was separated on a 5–20% gradient SDS-PAGE gel and transferred onto PVDF membrane (Millipore). Detection of HA-Ca_v_3.2 was performed using a primary rat monoclonal anti-HA antibody (1:1000, Roche) and secondary HRP-conjugated antibody (1:10,000, Jackson ImmunoResearch). Immunoreactive products were detected by enhanced chemiluminescence and analyzed using ImageJ software.

For co-immunoprecipitation, cell lysates containing GFP-tagged and Myc-tagged Ca_v_3.2 were incubated for 3 h with a biotinylated mouse monoclonal anti-Myc antibody (Santa Cruz Biotechnology), and then for 45 min with streptavidin beads (Invitrogen) at 4 °C, and washed with PBS/Tween-20 buffer. Beads were resuspended in Laemmli buffer and immunoprecipitation samples were separated on SDS-PAGE gel.

### Generation of human Ca_v_3.2 homology model

The homology model of the human Ca_v_3.2 channel was prepared using the Ca_v_3.1 structure as a template (PDB: 6KZO) in conjunction with Swiss-Model server (https://swissmodel.expasy.org/) [47]. Figures were prepared using Pymol (v2.2 Schrödinger, LLC.).

### Statistics

Data values are presented as mean ± SEM for *n* measurements. Statistical analysis was performed using GraphPad Prism 7. For datasets passing the D’Agostino & Pearson omnibus normality test, statistical significance was determined using either Student’s t-test or a Mann-Whitney test. Datasets were considered significantly different for *p* ≤ 0.05.

## Data Availability

The data used and/or analyzed during the current study are available from the corresponding author on reasonable request.

## Abbreviations

ALS: Amyotrophic lateral sclerosis
DRG: Dorsal root ganglia
GFP: Green fluorescent protein
*G*_max_: Maximal macroscopic conductance
MRI: Magnetic resonance imaging
PM: Pore mutant
*Q*_max_: Maximal charge movements
*Q*_rev_: Charge movements at the reversal calcium potential
WT: Wild-type
